# Health Literacy and Self‐Care Among Patients Living With Chronic Kidney Disease in a Low‐Resource Setting

**DOI:** 10.1002/nop2.70375

**Published:** 2025-11-12

**Authors:** Racheal Nakimuli Mwesigwa, Joyce Nankumbi, Tom Denis Ngabirano, Lydia Kabiri, Connie Olwit

**Affiliations:** ^1^ Mulago Hospital Kampala Uganda; ^2^ Department of Nursing Makerere University Kampala Uganda; ^3^ School of Nursing Augusta University Augusta Georgia USA

**Keywords:** chronic kidney disease, health literacy, low resource setting, self‐care

## Abstract

**Aim:**

The aim of this study was to determine whether health literacy predicts self‐careamong patients with chronic kidney disease (CKD) in a low‐resource setting.

**Design:**

The study was a cross‐sectional study conducted among 196 patients with CKD attending an urban facility in Kampala, Uganda.

**Methods:**

Data were collected using the adopted health literacy scale (HLS‐14 tool) and CKD self‐care questionnaire. Descriptive statistics and linear regression were used in the data analyses. The statistical significance level was set at *p* < 0.05.

**Results:**

The mean age of the participants was 47.2 (13.3) years. Most participants were male (60%), and about 72% were at stage 5 of CKD. Only 6% had health insurance whereas 20% were employed. The participants had a mean self‐care score of 65.39 (SD ±7.1) and a mean score of health literacy of 49.48 (SD ±11.2). Based on the bivariate analysis, self‐care was associated with sex (*p* = 0.005), employment status (*p* = 0.003) and health literacy (*p* = 0.005). Results from the multivariable regression model show that self‐care has a statistically significant positive association with health literacy (*β* = 0.167, *t* = 3.65, *p* ≤ 0.001) and employment (*β =* 4.45, *t* = 3.43, *p* ≤ 0.001) while controlling for other variables.

**Patient or Public Contribution:**

Our findings suggest that higher levels of health literacy are associated with improved self‐care practices which are crucial for managing CKD efficiently. Health education should be provided as early as possible. This work can serve as a basis for future related studies in low‐income settings.

## 1 Background

With an increasing prevalence of non‐communicable diseases in sub‐Saharan Africa by 67.0% between 1990 and 2017, there is an increasing challenge for health systems with insufficient data to navigate such challenges (Gouda et al. 2019). Noncommunicable diseases kill 41 million people each year, with 77% of these deaths occurring in low and middle‐income countries (WHO 2022). Chronic kidney disease (CKD) is one of the non‐communicable diseases in sub‐Saharan Africa, in addition to diabetes, cardiovascular diseases, cancers, and mental and substance use disorders. CKD is associated with poor quality of life, poor health outcomes, and high healthcare costs (WHO 2022). The prevalence of CKD is 10.7% in sub‐Saharan Africa (George et al. 2019; Matsha and Erasmus 2019) and 10.4% among men and 11.8% among women aged ≥ 20 years globally (Kovesdy 2022; Mills et al. 2015). A prevalence of 15.8% for stages 1–5 on the African continent was reported (Kaze et al. 2018). The prevalence of CKD in Uganda ranges between 2% and 7% (Kalyesubula et al. 2017; Muiru et al. 2020). The causes of CKD include glomerulonephritis, diabetes mellitus, hypertension, polycystic kidney disease, and gout (Tsai et al. 2021). With an estimation of about 4–9 million people requiring renal replacement therapy (RRT) globally, only 2.6 million are on dialysis, which implies over 2.3 million premature deaths as a result of access to RRT (Liyanage et al. 2015; Ogundele 2018). In addition, RRT as the treatment of choice including haemodialysis, peritoneal dialysis and renal transplant is unaffordable in our setting (Ogundele 2018); therefore it is necessary to explore feasible options including self‐management‐based interventions to prevent or slow the progression of the disease due to limited resources and a high cost of treatment.

Self‐management strategies have been identified as crucial in improving health outcomes, particularly for those with chronic diseases (Mackey et al. 2016; Ruiz et al. 2014) including CKD. Multiple patient characteristics including health literacy, have been associated with the development of self‐care behaviours (Mackey et al. 2016). This implies that patients have a very active role in their health care. This has been documented in several chronic diseases including diabetes, and cardiovascular diseases among others (Mackey et al. 2016). Health literacy (HL) is when one can apply any wellness information that was previously searched, obtained, and contemplated (Nutbeam 2008). This entails the ability to get, scrutinise and utilise information in day‐to‐day life in making decisions for the maintenance of good health (Berkman et al. 2010). HL is a chief factor in the care of patients with kidney diseases and contributes to CKD awareness and patient engagement in healthy behaviours (Stømer et al. 2020). Insufficient health literacy is not uncommon among patients with CKD. Low health literacy is associated with deteriorating kidney function (Fraser et al. 2013) and is a determinant of hospitalisation rates and mortality among CKD high‐risk patients (Griva et al. 2020). Patients with low levels of health literacy also practice sub‐optimal activities in the management of disease (Shah et al. 2021) and tend to experience unfavourable health consequences as they undertake high expenses (Devraj et al. 2015). Low health literacy can be concealed yet deplorable to patients, precipitating disgrace among patients that are uncertain about practising good self‐care behaviours (Mackey et al. 2016). In our setting, there are minimal studies that have documented HL, particularly for chronic diseases such as CKD; yet there is a need to identify areas for intervention regarding health literacy and self‐care. Such interventions can curb low health literacy and limit the negative effects of low health literacy on the development of CKD along the life course (do Amaral et al. 2021).

Self‐care refers to all events involving the patient's decision‐making in doing what is obliged for good health with the available resources to sustain life. Patients with chronic disease who engage in self‐care have elevated and improved health status (Donald et al. 2018). Such behaviours include physical activity, smoking cessation, decreased alcohol consumption, and sustaining a healthy BMI (Donald et al. 2018). Lifestyle changes among patients of CKD comorbid with hypertension include ‘Respecting low salt and eating a healthy diet’, ‘walking’, ‘non‐smoking and non‐alcohol’ and ‘Running’ (Mbabazi et al. 2022). High self‐care and disease knowledge scores are associated with a reduced likelihood of deterioration in kidney function, unlike low self‐care and disease knowledge scores (Tsai et al. 2021). An increase in kidney disease knowledge increases self‐care, and these are found to be predominant among patients with adequate health literacy (Schrauben et al. 2020). Patients who are more knowledgeable about renal disease have better overall CKD self‐care (Schrauben et al. 2020). Greater knowledge about CKD increases people's confidence and comfort when communicating with their healthcare providers, while limited knowledge disrupts this interaction because of the shame of not understanding the healthcare aspects (Shah et al. 2021). Interventions that put health literacy into consideration are crucial for better self‐care practices. Such approaches promote patient empowerment, improving adherence to self‐management strategies (Mackey et al. 2016). There is a paucity of information on health literacy, self‐care and associated factors among patients with CKD in Uganda. Therefore, this study aimed at determining whether health literacy predicts self‐care and their correlates among patients with CKD at an urban facility in Uganda.

## 2 Methods

### 2.1 Study Design

This was a cross‐sectional study that collected data from patients with CKD to examine the predictors of health literacy, self‐care and their association with socio‐demographic and health history factors. The Strengthening the Reporting of Observational Studies in Epidemiology (STROBE) guidelines were used to report the conduct and outcome of this study.

### 2.2 Participants and Settings

The study included adults with CKD patients attending a dialysis unit aged 18 years or older who are diagnosed with CKD stages 1–5 for 3 months or more. On average, about 45–60 dialysis sessions are carried out daily. This study was conducted at an urban facility in Kampala, Uganda. This is the biggest of the few facilities that exist in the country and offer haemodialysis. The unit exists at the national referral hospital and receives referrals from all over the country. The health facility offers services, including in and outpatient services, 24/7 emergency services, and outpatient specialised clinics, including dialysis, among others. Services provided to CKD patients include counselling. Health workers including doctors, nutritionists, and nurses provide health education. The dialysis unit receives about 40 patients per day and about 207 per week (Hospital Records, 2022). The outpatient clinic operates every Tuesday, attending to about 10 patients daily (Hospital Records, 2022).

### 2.3 Sample Size Considerations

A total of 196 participants were included in the study. This sample size was calculated based on the assumption that health literacy and self‐care behaviours are normally distributed. Due to a lack of information from previous studies to estimate the proportion with the outcome of interest, we assumed that 50% of the patients with CKD have low literacy levels and poor self‐care behaviours. With a sample of 196 patients, we were able to estimate the prevalence of low health literacy, and poor self‐care practices with 5% absolute precision and 95% confidence (Dhand and Khatkar 2014).

### 2.4 Measurements

Assessments were conducted using a researcher‐administered questionnaire. The questionnaire comprised three sections. In the first section, information on sociodemographic characteristics including age, sex, tribe, marital status, education, living situation, access to health care insurance, income, smoking, alcohol consumption and employment; health history including comorbidities, duration of CKD and stage, and duration on dialysis treatment; and anthropometric characteristics including weight, height, BMI and current medication were collected. The second and third sections collected information on HL and self‐care behaviours respectively.

### 2.5 Health Literacy

Health literacy was defined as the extent to which one can be able to search for, retrieve and contemplate any health information in deciding about their well‐being (Ratzan and Parker 2000). Health literacy was assessed using the 14‐item Health literacy scale (HLS‐14) which was validated and used in Japan. The Cronbach's alpha was 0.83, 0.85 and 0.76 for the functional, communicative and critical HL scores (Suka et al. 2013). The HLS‐14 was adopted in 2013 by Machi Suka of Jikei University School of Medicine with his team, and permission to use the tool was sought and granted. This tool was originally based on the HL scale specific to diabetic patients and was reported to have an overall Cronbach's alpha of 0.86 (Ishikawa et al. 2008). The health literacy tool comprises three sub‐scales; sub‐scale one focuses on functional health literacy (five items). Subscale two contains phrases about communicative health literacy (five items). Subscale three contains phrases about critical health literacy (four items). The responses to the HLS‐14 functional health literacy scale numbered 1 to 5 are rated using a five‐point Likert scale: ‘Strongly disagree’ (five points), ‘Disagree’ (four points), ‘Not sure’ (three points), ‘Agree’ (two points), ‘Strongly agree’ (one point). The responses to the HLS‐14 communicative and critical health literacy scales numbered 6 to 14 are rated: ‘Strongly disagree’ (one point), ‘Disagree’ (two points), ‘Not sure’ (three points), ‘Agree’ (four points), ‘Strongly agree’ (five points). Patients were asked to rate their health literacy from strongly agree to disagree strongly. Higher scores depict higher health literacy, and low scores depict lower health literacy. The lowest and the highest scores include 14 and 70 respectively. The score for health literacy was analysed as a continuous variable. The Cronbach's alpha in this study was 0.88.

### 2.6 Self‐Care

Self‐care was defined as activities of physical care and efforts made towards wellness in the day‐to‐day life of patients (Curtin et al. 2008). This was assessed using the chronic kidney disease self‐care (CKDSC) questionnaire. This tool was used in Taiwan (Wang et al. 2019) and Korea (Ahn et al. 2022) with a reported cronbach's alpha of 0.826 and 0.981 respectively (Ahn et al. 2022). The CKDSC is a 16‐item questionnaire with five items on medication adherence, four items on diet control, three items on exercise, two items on smoking behaviours, and two items on blood pressure monitoring. The CKDSC questionnaire items measure self‐care on a five‐point Likert scale (Wang et al. 2019). Responses numbered 1 to 5 are rated as ‘Strongly disagree’ (five points), ‘Disagree’ (four points), ‘Not sure’ (three points), ‘Agree’ (two points), and ‘Strongly agree’ (one point). Responses numbered 6 to 16 are rated as: ‘Strongly disagree’ (one point), ‘Disagree’ (two points), ‘Not sure’ (three points), ‘Agree’ (four points) and ‘Strongly agree’ (five points). Patients were asked to rate their self‐care from strongly agree to disagree strongly. Higher scores depicted higher self‐care, and low scores depicted lower self‐care. The minimum and maximum scores include 16 and 80, respectively. The median self‐care score for the current study population was used to determine the cutoffs. Earlier, a similar approach was used to categorise self‐care among patients with CKD in Taiwan (Tsai et al. 2021). The cronbach's alpha for this study was 0.77.

### 2.7 Data Collection

Study participants were recruited after obtaining permission from the facility and the unit Incharge. Eligible participants were recruited by the assistant nursing officer who triaged and registered the patients in the clinic and the outpatient department. The researcher sought consent from the eligible participants after providing information about the study. A convenient environment for the participant was identified and the questionnaire was completed within 30–40 min.

### 2.8 Data Analysis

Data analysis was performed using Statistical Package for Social Scientists (SPSS) version 26. Descriptive statistics were conducted for the characteristics of participants, health literacy and self‐care parameters.

**FIGURE 1:**
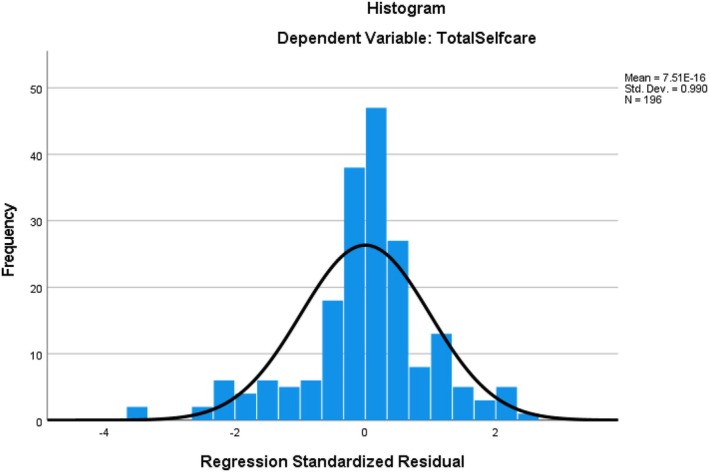
Histogram showing normal distribution of the dependent variable.

**FIGURE 2:**
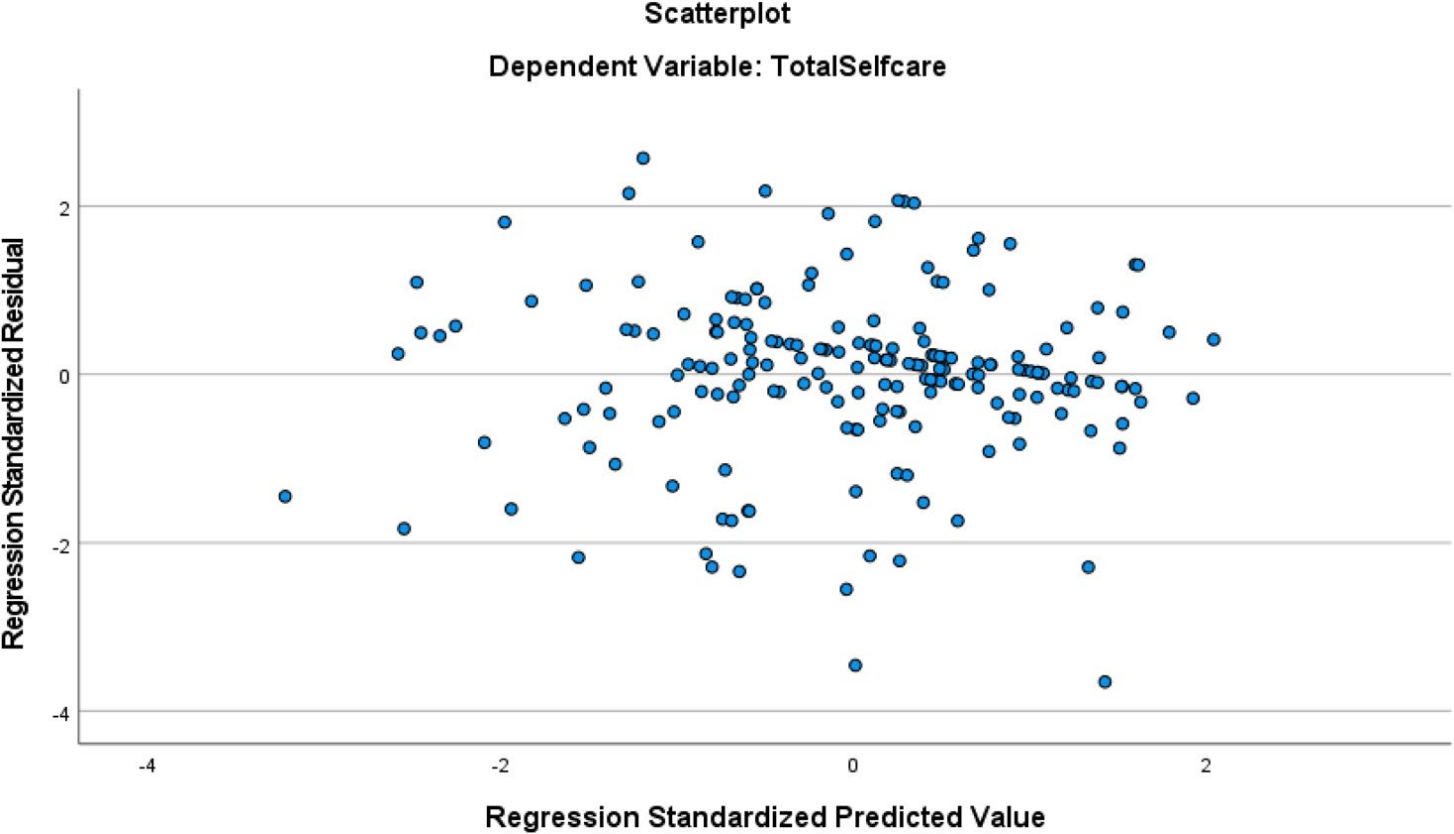
Scatterplot showing distribution of standardised residual.

The multivariable regression model was checked for linear regression assumptions and all assumptions were met. The outcome variable was normally distributed (Figure 1), Linearity was checked from a correlation matrix; all correlation coefficients were greater than zero, Independence (Durban‐Watson test was 1.84 which is between 1.5 and 2.5), homoscedasticity; the residues were spread somewhat evenly across the predicted values on the *X*‐axis (Figure 2), There was no multicollinearity (tolerance > 0.1, VIF < 10), no significant outliers (cook's *D* is 0.006 within ±3). Statistical significance was set at *p* < 0.05.

## 3 Results

### 3.1 Study Participants Characteristics

The mean age of the participants was 47.24 (13.3) years, ranging from 18 to 80 years. Nearly 60% of the participants were males. Three‐quarters of the participants 148 (75.5%) were married and half of the participants 99 (50.5%) completed secondary education (Table 1). The majority of the participants (72.4%) were in stage 5 of the disease. Only five participants (2.6%) reported having CKD for more than 5 years. More than two‐thirds of the participants 150 (76.5%) had been on dialysis for less than 12 months (Table 1).

**TABLE 1:** Sociodemographic characteristics of the participants.

Variables		*f* (%)	Mean (SD)
Age (years)	18–34	32 (16.3)	47.24 (13.27)
	35–49	81 (41.3)	
	50–64	64 (32.7)	
	> 64	19 (9.7)	
Tribe	Baganda	85 (43.3)	
	Banyankore	14 (7.1)	
	Basoga	14 (7.1)	
	Itesot	13 (6.6)	
	Others	70 (35.7)	
Sex	Male	117 (59.7)	
	Female	79 (40.3)	
Marital status	Single	41 (20.9)	
	Married	148 (75.5)	
	Widowed/divorced	7 (3.6)	
Education	No formal education/Primary	49 (25)	
	Secondary	99 (50.5)	
	Tertiary	48 (24.50)	
Living situation	Alone	7 (3.6)	
	Family/friends	189 (96.4)	
Health insurance	Insured	12 (6.1)	
	Not insured	184 (93.9)	
Income	≤ 1 million ugx	163 (83.2)	
	> 1 million ugx	33 (16.8)	
Employment status	Yes	39 (19.9)	
	No	157 (80.1)	
Smoking Status	Yes	2 (1.0)	
	No	194 (99.0)	
Alcohol consumption	Yes	1 (0.5)	
	No	195 (99.5)	
Duration of CKD (years)	< 1	121 (61.7)	
	1–4	70 (35.7)	
	≥ 5	5 (2.6)	
Duration on dialysis (years)	< 1	150 (76.5)	
	1–4	45 (23.0)	
	≥ 5	1 (0.5)	

Abbreviations: CKD, chronic kidney disease; SD, standard deviation.

### 3.2 Health Literacy of the Study Participants

Of the 196 participants recruited, only 50.5% of the participants had high health literacy scores. The overall mean HLS‐14 score was 49.78 (11.19) with scores ranging from 14 to 70. The mean subscale scores were: communicative health literacy 20.21 (4.57); critical health literacy 15.77 (3.86); and functional health literacy 13.80 (6.53). Of all the subscales, functional health literacy had the lowest mean score. The highest‐scored item on the functional health literacy scale was ‘The content is too difficult for me’, while the lowest‐scored items were ‘It takes a long time to read them’ and ‘I need someone to help me read them’. The communicative health literacy subscale had the highest mean score. The highest‐scored item on this scale was ‘I apply the obtained information to my daily life’, while the lowest was ‘I extract the information I want’ (Table 2).

**TABLE 2:** Chronic kidney disease health literacy scores.

	M (SD)	Range	Skewness
Overall score	49.78 (11.2)	14–70	−0.52
HL constructs			
Functional HL	13.80 (6.5)	5–25	0.29
Communicative HL	20.21 (4.6)	5–25	−1.39
Critical HL	15.77 (3.9)	4–20	−1.18

Abbreviations: HL, health literacy; SD, standard deviation.

### 3.3 Self‐Care Behaviours of the Study Participants

The self‐care behaviours include medication adherence, diet control, exercise, smoking behaviour and blood pressure measurement. The overall mean CKDSC score was 65.39 (7.1) with a range between 42 and 80. The mean sub‐scale scores were: medication adherence 22.88 (3.2); diet control 17.74 (3.6); exercise 5.59 (3.7); smoking behaviour 9.81 (0.8); and blood pressure measurement 9.40 (1.44). The medication adherence scale had the highest score for self‐care (Table 3). The highest and lowest scored items on this scale were ‘I myself may change prescribed drug dosage’ and ‘I may change the prescribed dosing time’, respectively. The exercise scale had the lowest score for self‐care. The lowest and highest total scores on the scale were 3 and 15, respectively. The highest and lowest scored items on this scale were ‘I may still try to work out to keep my kidney disease under control whenever I do not want to do exercise’ and ‘I may still try to take time out of my busy schedule to work out’, respectively. Good self‐care was defined as scores ≥ 67 while low self‐care was defined as scores < 67 (Tsai et al. 2021). Slightly more than half 53.1% had self‐care scores above the median indicating good self‐care behaviours.

**TABLE 3:** Chronic kidney disease self‐care scores.

	M (SD)	Range	Skewness
Overall score	65.39 (7.1)	42–80	−0.75
Self‐care constructs			
Medication adherence	22.88 (3.2)	9–25	−2.06
Diet control	17.74 (3.6)	4–20	−2.29
Exercise	5.59 (3.7)	3–15	1.44
Smoking behaviour	9.8 (0.8)	5–10	−4.61
Blood monitoring	9.39 (1.4)	2–10	−3.08

Abbreviation: SD, standard deviation.

### 3.4 Factors Associated With Self‐Care Behaviour

In the univariate model, self‐care scores were significantly associated with sex (*β* = 0.126, *p* ≤ 0.005), employment status (*β* = 0.244, *p* ≤ 0.003) and health literacy scores (*β* = 0.126, *p* ≤ 0.005). In the multivariate model, after controlling for other variables, results showed that self‐care has a statistically significant positive association with health literacy (*β* = 0.167, *t* = 3.65, *p* ≤ 0.001) and employment (*β =* 4.45, *t* = 3.43, *p* ≤ 0.001) (Table 4).

**TABLE 4:** Results from univariate and multivariable regression models for examining associated with CKD self‐care score.

Variable	Univariate model		*β*	Multivariable model	
	*β*	*p*		*p*	*t*
Age (years)	0.004	0.908	0.04	0.295	1.051
Sex	0.126	0.005**	0.543	0.60	0.525
Marital status	0.244	0.837			
Employment status	3.82	0.003**	4.45	< 0.001***	3.435
Duration of CKD diagnosis	−0.018	0.577			
CKD stages	−0.065	0.976			
Living status (social support)	−0.185	0.946			
Health literacy	0.126	0.005**	0.167	< 0.001***	3.657

*Note:* *R* ^2^ = 0.109, *f* (df1, df2) = 5.851 (4,191).

Abbreviation: CKD, chronic kidney disease.

** statistically significant.

*** statistically significant with *p* values less than 0.001.

## 4 Discussion

The results of this study indicated that there was a positive association between health literacy and self‐care behaviours. The results showed that 50.1% of the participants had high levels of health literacy. Given, that patients receive health education sessions from the facility and have a chronic condition, it creates awareness for the patients. At the same time, most study participants had a relatively higher level of education and hence better knowledge or awareness of the disease. This promotes health literacy since patient knowledge and understanding contribute to health literacy (Reisi et al. 2017). However, findings from this study are lower than those from a study done in Singapore where 68.9% of the patients had adequate health literacy (Ho et al. 2022) and a study carried out in the USA which reported 82.3% of the participants having sufficient health literacy (Schrauben et al. 2020). In another study carried out in San Francisco, 74% of the participants had high health literacy (Wong et al. 2018). The differences in findings could be attributed to exposures or sources of information readily available to the patients. Differences in the health care arrangements or systems where the patients are more empowered with information about their condition compared to a low‐resource setting like Uganda. On the other hand, the level of health literacy was, higher compared to that reported in a study carried out in Taiwan, where 46.63% of the participants were reported to have sufficient health literacy (Yu et al. 2021). Similarly, a study conducted in New Mexico (Devraj et al. 2015) and Brazil (Moraes et al. 2017) reported 36.7% and generally low health literacy levels. The difference in findings could be due to the setting of these studies in countries of low‐income levels and hence decreased investment in the health sector to promote health education to improve health literacy. In this study, the communicative health literacy scale had the highest scores of health literacy with the item ‘I tell my opinion about my illness to my doctor, family or friend’ having the highest number of responses. This could be because the patients tend to seek information from different sources about their health and they tend to share their opinions to obtain feedback. These findings are similar to those from a study conducted in Norway, where ‘feeling understood and supported by healthcare providers’ and ‘ability to actively engage with health‐care providers’ had the highest number of responses from the HLQ (Stømer et al. 2019). The similarity in findings could be due to comparable expectations of patients with CKD.

In this study, 53.1% of the participants had high levels of self‐care. The mean CKDSC score in this study was 65.39 ± 7.11, which was similar to that reported by Wang et al., in a study on self‐care among patients with CKD in Taiwan (Wang et al. 2019). However, their scores for diet control, smoking and blood pressure control were slightly lower than the scores in this study. The medication adherence scale had the highest scores on the CKDSC tool. This was distinct from the findings in a study carried out in the USA (Wong et al. 2018) where less than a third of the participants had high medication adherence scores. This indicates lower levels of medication adherence in our setting and many factors such as the cost of the medication, and inaccessibility can contribute to this deficiency. The results from this study conflicted with those from a study carried out in Rwanda (Mbabazi et al. 2022) where participants were reported to forget to take their medications and ceased taking them without medical advice. The findings from this study are also contrary to those from a study carried out in Thailand (Shayakul et al. 2022) which reported that most of the patients were not taking their medications as prescribed, forgot to take their medications and adjusted their dosage regimens. The differences can be attributed to the late stages of CKD in which most participants were. This means that the participants endeavour to take medications to maintain their health. In our study, the lowest mean CKDSC scores were from the scale of exercise. This could be due to the low energy levels of the patients owing to the deficiency of renal erythropoietin, and hence anemia (Wang et al. 2019).

The present findings identified factors that are associated with self‐care behaviour including sex, employment and health literacy. Such results may not be surprising as women tend to engage more proactively in their health management thus better self‐care practices than men. Women are better health care seekers than men. These results are not different from a study conducted in Ghana that looked at the correlates of sociodemographics and behavioural correlates of health literacy between men and women (Amoah and Phillips 2020). The association between health literacy and self‐care behaviour indicates that it is important to continue providing health education to patients with CKD if we are to continue advocating for patients and improve the quality of life in CKD.

### 4.1 Strength and Limitations

One of the strengths of this study is that it is one of a kind. This sets a platform for other researchers in our setting. Secondly, the sample size was considered adequate. However, we might have dealt with a sample that is already engaged with health care. At the same time, having used a self‐ reported instrument to measure HL and self‐care practices, the results of the study should be interpreted with caution. Some participants may probably have exaggerated or underreported some of the tool items. Future studies may focus on the psychometric properties of the tools translated into the local languages. Causal inferences cannot be drawn from the findings as the analyses were entirely based on a cross‐sectional design. Nonetheless, the research instruments were robust and the findings are mostly consistent with results from other studies.

## 5 Conclusion

Our study has identified a significant correlation between health literacy, self‐care behaviours and employment status among patients with CKD. Our findings suggest that higher levels of health literacy are associated with improved self‐care practices which are crucial for managing CKD efficiently. Additionally, the analysis revealed that sex and employment status play important roles in shaping self‐care behaviours, indicating that these factors should be considered when developing targeted interventions. Health education should be offered as early as possible to avoid the advanced stages of the disease. This work can serve as a basis for future related studies in low‐income settings.

## Ethics Statement

This study was approved. ‘REDACTED’ approval was also obtained from the health facility. Participants were included in the study after informed consent.

## Conflicts of Interest

The authors declare no conflicts of interest.

## Data Availability

The data that support the findings of this study are available on request from the corresponding author. The data are not publicly available due to privacy or ethical restrictions.
